# Single-step genomic BLUP with genetic groups and automatic adjustment for allele coding

**DOI:** 10.1186/s12711-022-00721-x

**Published:** 2022-06-02

**Authors:** Ismo Strandén, Gert P. Aamand, Esa A. Mäntysaari

**Affiliations:** 1 Natural Resources Institute Finland (Luke), Jokioinen, Finland; 2 Nordic Cattle Genetic Evaluation (NAV), Aarhus, Denmark

## Abstract

**Background:**

Genomic estimated breeding values (GEBV) by single-step genomic BLUP (ssGBLUP) are affected by the centering of marker information used. The use of a fixed effect called J factor will lead to GEBV that are unaffected by the centering used. We extended the use of a single J factor to a group of J factors.

**Results:**

J factor(s) are usually included in mixed model equations (MME) as regression effects but a transformation similar to that regularly used for genetic groups can be applied to obtain a simpler MME, which is sparser than the original MME and does not need computation of the J factors. When the J factor is based on the same structure as the genetic groups, then MME can be transformed such that coefficients for the genetic groups no longer include information from the genomic relationship matrix. We illustrate the use of J factors in the analysis of a Red dairy cattle data set for fertility.

**Conclusions:**

The GEBV from these analyses confirmed the theoretical derivations that show that the resulting GEBV are allele coding independent when a J factor is used. Transformed MME led to faster computing time than the original regression-based MME.

## Background

Single-step genomic BLUP (ssGBLUP) [1, 2] requires that the pedigree and genomic relationship matrices are compatible [3]. Two measures of similarity have been considered [4]: averages of diagonal and all elements. These two statistics are affected by the completeness of pedigree information. In pedigree-based animal model evaluations, incomplete pedigree information is often modeled by genetic groups [5]. Elements of the genomic relationship matrix are typically computed using centered and scaled marker genotypes [6]. Both centering and scaling often depend on allele frequencies and are affected by the available animal genotypes and, when pedigree information is used in the allele frequency estimation, by the completeness of the pedigree. Thus, incomplete information can affect both the pedigree and the genomic relationship matrix.

Fernando et al. [7] proposed a marker-based single-step model using Bayesian regression. When all the variance components are known, this model, hereafter called ssSNPBLUP, is equivalent to ssGBLUP. In their ssSNPBLUP, the genomic estimated breeding values (GEBV) are made independent of the allele frequencies that are used for centering marker genotypes by a regression effect, hereafter called J factor, which adjusts the breeding values to the appropriate level [8]. This is similar to a simple genomic model without pedigree information, often called SNP-BLUP, where the marker effect solutions are independent of allele coding but, for the GEBV to be independent of allele coding, their level needs to be adjusted by a general mean [9]. Thus, in both ssGBLUP/ssSNPBLUP and SNP-BLUP, estimating a fixed effect and adding its solution to the estimated genetic values allows the calculation of GEBV that are independent of the allele coding or centering of the genotypes used. Fitting a J factor in a single-step model has improved prediction accuracy when selection acted on the evaluated trait [8]. Furthermore, the use of a J factor has been observed to increase accuracy and lower bias in the analysis of simulated data [10].

In practice, the pedigrees are incomplete and animals with information descend from different base populations. For the pedigree-based animal models, Thompson [11] suggested the use of parent genetic groups to account for differences in genetic levels of the base populations. The genetic groups were modeled by regression coefficients. The resulting EBV were functions of genetic group solutions and additive genetic effects, similar to the J factor being part of the breeding value. Quaas and Pollak [12] showed that the so-called QP transformation can be used to model the genetic groups as unknown parent groups (UPG) in mixed model equations (MME). The use of the QP transformation allows a computationally efficient approach to include the regression effects of genetic groups in MME by augmenting the UPG into the inverse relationship matrix. Furthermore, the breeding values from MME by the QP transformation include the effect of genetic group information and, hence, there is no need to add the group effect solutions afterward to the estimated genetic effects. Misztal et al. [13] noted the computational difficulties of full QP transformation in ssGBLUP MME and discussed alternative approaches to fit genetic groups. Matilainen et al. [14] implemented the full QP transformation in multiple trait ssGBLUP of national dairy cattle fertility data with 11 traits. They observed that the full QP transformation guaranteed good convergence of the iterative method when solving the MME.

In this study, we use the J factor in the original ssGBLUP model and extend the J factor approach to include the same structure as for the genetic groups. We derive simple MME by applying a QP-like transformation to the J factor and consider computational aspects of genomic relationship matrices in the transformed MME. We illustrate the effects of including the genetic groups and extended J factors on ssGBLUP using a Nordic Red dairy cattle fertility data set.

## Methods

### Single-step GBLUP model with genetic groups and J factors

We consider a single-trait single-step GBLUP (ssGBLUP) model:

1 $$\mathbf{y}=\mathbf{X}\mathbf{b}+\mathbf{W}\mathbf{J}\mathbf{c}+\mathbf{W}\mathbf{Q}\mathbf{g}+\mathbf{W}\mathbf{a}+\mathbf{e},$$

where $\mathbf{b}$ is a vector of fixed effects, $\mathbf{c}$ is an $s$ by 1 vector of fixed genetic centering, i.e., J factor, regression effects [7], $\mathbf{J}$ is a $q$ by $s$ matrix of known coefficients, $\mathbf{g}$ is an $r$ by 1 vector of random genetic group regression effects, $\mathbf{Q}$ is a $q$ by $r$ matrix of known coefficients, $\mathbf{a}$ is a $q$ by 1 vector of random additive genetic effects, and $\mathbf{e}$ is a random residual vector. Matrix $\mathbf{X}$ relates fixed effects $\mathbf{b}$ and matrix $\mathbf{W}$ relates effects of centering $\mathbf{J}\mathbf{c}$, genetic groups $\mathbf{Q}\mathbf{g}$ and additive genetics $\mathbf{a}$ to appropriate observations in vector $\mathbf{y}$. Matrix $\mathbf{J}$ has coefficients of genetic proportions in the $s$ centering groups for the genotyped animals but imputed proportions for the non-genotyped animals. This matrix will be described below. The estimated fixed effects $\mathbf{c}$ allow to compute GEBV that will be unaffected by the centering of marker genotypes used when building the genomic relationship matrix, i.e., the GEBV will be free from the used allele coding. We assume $Var\left(\mathbf{a}\right)=\mathbf{H}{\sigma }_{a}^{2}$ and $Var\left(\mathbf{e}\right)=\mathbf{R}$. In the following derivations, we assume that the genetic groups are random with an expectation of zero and variance $\mathbf{S}$. When fixed genetic groups are assumed, the resulting MME (below Eqs. (2–6)) contain neither $\mathbf{S}$ nor ${\mathbf{S}}^{-1}$.

Matrix ${\mathbf{H}}^{-1}$ in the MME of ssGBLUP is according to [1, 2]:

$${\mathbf{H}}^{-1}={\mathbf{A}}^{-1}+\left[\begin{array}{cc}{\mathbf{0}}& {\mathbf{0}}\\ {\mathbf{0}}& {\mathbf{G}}^{-1}-{\mathbf{A}}_{22}^{-1}\end{array}\right],$$

where $\mathbf{A}$ is the full pedigree relationship matrix, $\mathbf{G}$ is the genomic relationship matrix, and ${\mathbf{A}}_{22}$ is the pedigree-based relationship matrix of the genotyped animals. The genomic relationship matrix can be formed, for example, as $\mathbf{G}=\mathbf{Z}{\mathbf{D}}^{-1}\mathbf{Z}\mathbf{^{\prime}}$, where $\mathbf{Z}=\mathbf{M}-\mathbf{P}$ is a (centered) marker matrix of size $n$ by $m$ and $\mathbf{D}$ is a diagonal scaling matrix [6]. Each genotype value in the marker genotype matrix $\mathbf{M}$ is the number of alleles, with a value of 0 when the individual is homozygous for the first allele, 1 when the individual is heterozygous, and 2 when the individual is homozygous for the second allele. Matrix $\mathbf{D}$ is a diagonal scaling matrix. For example, the so-called VanRaden method 1 has $\mathbf{D}=k\mathbf{I}$, where $k=\sum_{l=1}^{m}2{p}_{l}\left(1-{p}_{l}\right)$ and ${p}_{l}$ is the (base) population allele frequency for marker $l$. Here, we assume the $\mathbf{Z}{\mathbf{D}}^{-1}\mathbf{Z}\mathbf{^{\prime}}$ matrix to be non-singular but the following derivations allow more general definitions of the $\mathbf{G}$ matrix, and we will consider them later.

Values in the centering matrix $\mathbf{P}=\mathbf{1}\mathbf{v}\mathbf{^{\prime}}$ often depend on the allele frequencies of the markers. For example, $\mathbf{v}=2\mathbf{p}\mathbf{^{\prime}}$ where $\mathbf{p}$ is an $m$ by 1 vector of base population allele frequencies [9]. Fernando et al. [7] proposed to include a fixed regression effect in ssSNPBLUP such that the GEBV are unaffected by the chosen centering matrix $\mathbf{P}$. They defined $$\mathbf{J}=\left[\begin{array}{c}-{\mathbf{A}}_{12}{\mathbf{A}}_{22}^{-1}\mathbf{1}\\ \mathbf{-1}\end{array}\right]$$ as a vector having minus one for the genotyped animals and $-{\mathbf{A}}_{12}{\mathbf{A}}_{22}^{-1}\mathbf{1}$ for the non-genotyped animals where ${\mathbf{A}}_{12}$ is the pedigree-based relationship matrix between the non-genotyped (subscript 1) and genotyped (subscript 2) animals. A random J factor approach was presented for ssGBLUP in Vitezica et al. [3] and will be considered in the "Discussion" Section.

The ssSNPBLUP model by Fernando et al. [7] is a model equivalent to the ssGBLUP Model Eq. (1). Thus, following Fernando et al. [7], GEBV in Model Eq. (1) are computed as ${\widehat{\mathbf{a}}}_{d}=\mathbf{J}\widehat{\mathbf{c}}+\mathbf{Q}\widehat{\mathbf{g}}+\widehat{\mathbf{a}}$, i.e., the J factor and the genetic groups are added to the additive genetic effects. GEBV ${\widehat{\mathbf{a}}}_{d}$ are independent of the centering of marker genotypes used, i.e., allele coding, due to the presence of the fixed J factor solutions $\mathbf{J}\widehat{\mathbf{c}}$. In ssSNPBLUP, the marker genotypes are used as regression coefficients where the marker genotypes for the non-genotyped animals are imputed from the genotyped animals using the linear imputation formula ${\mathbf{A}}_{12}{\mathbf{A}}_{22}^{-1}\mathbf{M}$. The coefficients ${\mathbf{A}}_{12}{\mathbf{A}}_{22}^{-1}$ in the imputation formula are used in the fixed J factor to “impute” the general mean from the genotyped animals to the non-genotyped animals. Consequently, any changes in the centering of the genotypes will change the additive genetic effect estimates $\widehat{\mathbf{a}}$ but changes due to the J factor estimates $\mathbf{J}\widehat{\mathbf{c}}$ allow the GEBV ${\widehat{\mathbf{a}}}_{d}$ to remain unchanged. This is like any linear model that has a fixed general mean, a linear shift in the regression coefficients will change the general mean estimate but lead to the same predicted observations as shown for SNP-BLUP in [9]. The independence of allele coding can be proved formally by generalizing the derivations for SNP-BLUP in [9]. The allele coding independence will also be realized in ssGBLUP, because ssSNPBLUP and ssGBLUP are equivalent.

We generalize the fixed J factor approach from a single regression effect to $s$ regression effects that may depend on the pedigree structure or predefined group status such as birth year or breed. Let the coefficient matrix $\mathbf{J}$ of the regression effect $\mathbf{c}$ be minus one times matrix ${\mathbf{Q}}_{\mathrm{c}}$ for the genotyped animals and $-{\mathbf{A}}_{12}{\mathbf{A}}_{22}^{-1}{\mathbf{Q}}_{\mathrm{c}}$ for the non-genotyped animals: $$\mathbf{J}=\left[\begin{array}{c}-{\mathbf{A}}_{12}{\mathbf{A}}_{22}^{-1}\\ -\mathbf{I}\end{array}\right]{\mathbf{Q}}_{\mathrm{c}}$$ where ${\mathbf{Q}}_{\mathrm{c}}$ is an ${n}_{g}$ by $s$ matrix having coefficients for the genotyped animals in the J factor groups and ${n}_{g}$ is the number genotyped animals. We assume that the sums of the rows of the ${\mathbf{Q}}_{\mathrm{c}}$ matrix equal 1, i.e., ${\mathbf{Q}}_{\mathrm{c}}\mathbf{1}=\mathbf{1}$, and every element in ${\mathbf{Q}}_{\mathrm{c}}$ is within the interval [0,1]. The generalization from a single to multiple J factors makes the need to account for differences in centering the genotypes between genotyped individuals simple. Explicit centering of the genotype matrix $\mathbf{M}$ using the ${\mathbf{Q}}_{\mathrm{c}}$ matrix, i.e., $\mathbf{Z}=\mathbf{M}-{2\mathbf{Q}}_{\mathrm{c}}{\mathbf{P}}_{\mathrm{c}}\boldsymbol{^{\prime}}$, where ${\mathbf{P}}_{\mathrm{c}}$ is an $m$ by $s$ matrix that has allele frequencies in the $s$ groups for the $m$ markers, becomes void using the multiple group J factor by the ${\mathbf{Q}}_{\mathrm{c}}$ matrix and follows from generalizing the development of Fernando et al. [7] and Strandén and Christensen [9]. For example, when the ${\mathbf{Q}}_{\mathrm{c}}$ matrix has breed proportions, the use of breed-wise allele frequencies for centering in the genomic relationship matrix [15] will give the same GEBV as those that use an allele frequency of 0.5 for all markers provided the same scaling is used.

Rows in the ${\mathbf{Q}}_{\mathrm{c}}$ coefficients matrix can have fractions of the base group proportions for the genotyped animal, which are calculated using pedigree information similarly to the coefficients in the ${\mathbf{Q}}_{2}$ matrix for the genotyped animals in the $\mathbf{Q}$ matrix for the unknown genetic groups. The J factor effects become confounded with the genetic group effects when ${\mathbf{Q}}_{\mathrm{c}}$ equals ${\mathbf{Q}}_{2}$, and all phenotyped animals have been genotyped. When the number of phenotyped animals without genotype information is small, there may be a situation close to collinearity with the genetic group and J factor effects since these effects will try to model the same effect. This is unlikely in many current breeding populations with long recording history and with many phenotyped animals without genotype information. However, some new traits such as greenhouse gas emission measurements have been recorded only recently and are likely to be from genotyped animals only. In the case when almost all the phenotyped animals have been genotyped, the J factor effect could be treated as operationally random. Otherwise, the J factor would be inseparable from the overall mean and the results may be meaningless. However, the ${\mathbf{Q}}_{\mathrm{c}}$ and ${\mathbf{Q}}_{2}$ matrices do not need to be the same. For example, the ${\mathbf{Q}}_{2}$ matrix can have genetic groups based on breed, birth year, country of origin, and sex but the ${\mathbf{Q}}_{\mathrm{c}}$ matrix can have fewer classes due to a pedigree that traces back far with distinct sub-populations, which can lead to the J factor coefficients in the ${\mathbf{A}}_{12}{\mathbf{A}}_{22}^{-1}{\mathbf{Q}}_{2}$ matrix for some genetic groups to be zero or close to zero. In the extreme, when ${\mathbf{Q}}_{\mathrm{c}}$ equals $\mathbf{1}$, our generalization reduces to the J factor in Fernando et al. [7].

### Transforming mixed model equations

MME for the ssGBLUP Model Eq. (1) are:

2 $$\left[\begin{array}{cccc}{\mathbf{X}}^{\mathrm{\prime}}{\mathbf{R}}^{-1}\mathbf{X}& {\mathbf{X}}^{\mathrm{\prime}}{\mathbf{R}}^{-1}\mathbf{W}\mathbf{J}& {\mathbf{X}}^{\mathrm{\prime}}{\mathbf{R}}^{-1}\mathbf{W}\mathbf{Q}& {\mathbf{X}}^{\mathrm{\prime}}{\mathbf{R}}^{-1}\mathbf{W}\\ {\mathbf{J}}^{\mathrm{\prime}}{\mathbf{W}}^{\mathrm{\prime}}{\mathbf{R}}^{-1}\mathbf{X}& {\mathbf{J}}^{\mathrm{\prime}}{\mathbf{W}}^{\mathrm{\prime}}{\mathbf{R}}^{-1}\mathbf{W}\mathbf{J}& {\mathbf{J}}^{\mathrm{\prime}}{\mathbf{W}}^{\mathrm{\prime}}{\mathbf{R}}^{-1}\mathbf{W}\mathbf{Q}& {\mathbf{J}}^{\mathrm{\prime}}{\mathbf{W}}^{\mathrm{\prime}}{\mathbf{R}}^{-1}\mathbf{W}\\ {\mathbf{Q}}^{\mathrm{\prime}}{\mathbf{W}}^{\mathrm{\prime}}{\mathbf{R}}^{-1}\mathbf{X}& {\mathbf{Q}}^{\mathrm{\prime}}{\mathbf{W}}^{\mathrm{\prime}}{\mathbf{R}}^{-1}\mathbf{W}\mathbf{J}& {\mathbf{Q}}^{\mathrm{\prime}}{\mathbf{W}}^{\mathrm{\prime}}{\mathbf{R}}^{-1}\mathbf{W}\mathbf{Q}+{\mathbf{S}}^{-1}& {\mathbf{Q}}^{\mathrm{\prime}}{\mathbf{W}}^{\mathrm{\prime}}{\mathbf{R}}^{-1}\mathbf{W}\\ {\mathbf{W}}^{\mathrm{\prime}}{\mathbf{R}}^{-1}\mathbf{X}& \mathbf{W}\mathrm{^{\prime}}{\mathbf{R}}^{-1}\mathbf{W}\mathbf{J}& \mathbf{W}\mathrm{^{\prime}}{\mathbf{R}}^{-1}\mathbf{W}\mathbf{Q}& \mathbf{W}\mathrm{^{\prime}}{\mathbf{R}}^{-1}\mathbf{W}+{\mathbf{H}}^{-1}{\upsigma }_{a}^{-2}\end{array}\right]\left[\begin{array}{c}\widehat{\mathbf{b}}\\ \widehat{\mathbf{c}}\\ \widehat{\mathbf{g}}\\ \widehat{\mathbf{a}}\end{array}\right]=\left[\begin{array}{c}\mathbf{X}\mathrm{^{\prime}}{\mathbf{R}}^{-1}\mathbf{y}\\ \mathbf{J}\mathrm{^{\prime}}\mathbf{W}\mathrm{^{\prime}}{\mathbf{R}}^{-1}\mathbf{y}\\ \mathbf{Q}\mathrm{^{\prime}}\mathbf{W}\mathrm{^{\prime}}{\mathbf{R}}^{-1}\mathbf{y}\\ \mathbf{W}\mathrm{^{\prime}}{\mathbf{R}}^{-1}\mathbf{y}\end{array}\right]$$

After solving the MME Eq. (2), the estimates of the breeding values are ${\widehat{\mathbf{a}}}_{d}=\mathbf{J}\widehat{\mathbf{c}}+\mathbf{Q}\widehat{\mathbf{g}}+\widehat{\mathbf{a}}$ [7, 11]. The QP transformation [3, 12] of MME Eq. (2) will provide MME where the breeding values ${\widehat{\mathbf{a}}}_{d}$ are estimated explicitly. Let $$\mathbf{P}=\left[\begin{array}{cccc}\mathbf{I}& \mathbf{0}& \mathbf{0}& \mathbf{0}\\ \mathbf{0}& \mathbf{I}& \mathbf{0}& \mathbf{0}\\ \mathbf{0}& \mathbf{0}& \mathbf{I}& \mathbf{0}\\ \mathbf{0}& \mathbf{J}& \mathbf{Q}& \mathbf{I}\end{array}\right]$$, $${\mathbf{P}}^{-1}=\left[\begin{array}{cccc}\mathbf{I}& \mathbf{0}& \mathbf{0}& \mathbf{0}\\ \mathbf{0}& \mathbf{I}& \mathbf{0}& \mathbf{0}\\ \mathbf{0}& \mathbf{0}& \mathbf{I}& \mathbf{0}\\ \mathbf{0}& -\mathbf{J}& -\mathbf{Q}& \mathbf{I}\end{array}\right]$$, and $$\widehat{\mathbf{v}}=\left[\begin{array}{c}\widehat{\mathbf{b}}\\ \widehat{\mathbf{c}}\\ \widehat{\mathbf{g}}\\ \widehat{\mathbf{a}}\end{array}\right]$$. The solution vector of all unknowns is $${\widehat{\mathbf{v}}}_{d}=\left[\begin{array}{c}\widehat{\mathbf{b}}\\ \widehat{\mathbf{c}}\\ \widehat{\mathbf{g}}\\ {\widehat{\mathbf{a}}}_{d}\end{array}\right]=\mathbf{P}\widehat{\mathbf{v}}$$, where the left-hand side has the breeding value estimates ${\widehat{\mathbf{a}}}_{d}$ calculated as linear function of the J factor, genetic group and genetic effect solutions. Let $\mathbf{C}$ and $\mathbf{r}$ be the coefficient matrix and the right-hand side vector in MME Eq. (2), respectively. In the QP transformation, the MME are transformed to be ${\left({\mathbf{P}}^{-1}\right)}^{{\prime}}\mathbf{C}{\mathbf{P}}^{-1}{\widehat{\mathbf{v}}}_{\mathrm{d}}={\left({\mathbf{P}}^{-1}\right)}^{{\prime}}\mathbf{r}$. MME of the QP transformed ssGBLUP are:

3 $$\left[\begin{array}{cccc}{\mathbf{X}}^{\mathrm{\prime}}{\mathbf{R}}^{-1}\mathbf{X}& {\mathbf{0}}& {\mathbf{0}}& {\mathbf{X}}^{\mathrm{\prime}}{\mathbf{R}}^{-1}\mathbf{W}\\ {\mathbf{0}}& {\mathbf{J}}^{\mathrm{\prime}}{\mathbf{H}}^{-1}\mathbf{J}{\upsigma }_{a}^{-2}& {\mathbf{J}}^{\mathrm{\prime}}{\mathbf{H}}^{-1}\mathbf{Q}{\upsigma }_{a}^{-2}& -{\mathbf{J}}^{\mathrm{\prime}}{\mathbf{H}}^{-1}{\upsigma }_{a}^{-2}\\ {\mathbf{0}}& {\mathbf{Q}}^{\mathrm{\prime}}{\mathbf{H}}^{-1}\mathbf{J}{\upsigma }_{a}^{-2}& {\mathbf{Q}}^{\mathrm{\prime}}{\mathbf{H}}^{-1}\mathbf{Q}{\upsigma }_{a}^{-2}+{\mathbf{S}}^{-1}& -{\mathbf{Q}}^{\mathrm{\prime}}{\mathbf{H}}^{-1}{\upsigma }_{a}^{-2}\\ {\mathbf{W}}^{\mathrm{\prime}}{\mathbf{R}}^{-1}\mathbf{X}& -{\mathbf{H}}^{-1}\mathbf{J}{\upsigma }_{a}^{-2}& -{\mathbf{H}}^{-1}\mathbf{Q}{\upsigma }_{a}^{-2}& {\mathbf{W}}^{\mathrm{\prime}}{\mathbf{R}}^{-1}\mathbf{W}+{\mathbf{H}}^{-1}{\upsigma }_{a}^{-2}\end{array}\right]\left[\begin{array}{c}\widehat{\mathbf{b}}\\ \widehat{\mathbf{c}}\\ \widehat{\mathbf{g}}\\ {\widehat{\mathbf{a}}}_{d}\end{array}\right]=\left[\begin{array}{c}\mathbf{X}\mathrm{^{\prime}}{\mathbf{R}}^{-1}\mathbf{y}\\ {\mathbf{0}}\\ {\mathbf{0}}\\ \mathbf{W}\mathrm{^{\prime}}{\mathbf{R}}^{-1}\mathbf{y}\end{array}\right].$$

The term ${\mathbf{H}}^{-1}\mathbf{J}$ in the MME Eq. (3) can be simplified. First, note that:

$${\mathbf{A}}^{-1}\mathbf{J}=\left[\begin{array}{cc}{\mathbf{A}}^{11}& {\mathbf{A}}^{12}\\ {\mathbf{A}}^{21}& {\mathbf{A}}^{22}\end{array}\right]\left[\begin{array}{c}-{\mathbf{A}}_{12}{\mathbf{A}}_{22}^{-1}\\ -\mathbf{I}\end{array}\right]{\mathbf{Q}}_{\mathrm{c}}=\left[\begin{array}{cc}{\mathbf{A}}^{11}& {\mathbf{A}}^{12}\\ {\mathbf{A}}^{21}& {\mathbf{A}}^{22}\end{array}\right]\left[\begin{array}{c}{\left({\mathbf{A}}^{11}\right)}^{-1}{\mathbf{A}}^{12}\\ -\mathbf{I}\end{array}\right]{\mathbf{Q}}_{\mathrm{c}}=\left[ \begin{array}{c}{\mathbf{0}}\\ {\mathbf{A}}^{21}{\left({\mathbf{A}}^{11}\right)}^{-1}{\mathbf{A}}^{12}-{\mathbf{A}}^{22}\end{array}\right]{\mathbf{Q}}_{\mathrm{c}}=\left[\begin{array}{c}{\mathbf{0}}\\ -{\mathbf{A}}_{22}^{-1}\end{array}\right]{\mathbf{Q}}_{\mathrm{c}},$$

because ${\mathbf{A}}_{12}{\mathbf{A}}_{22}^{-1}=-{\left({\mathbf{A}}^{11}\right)}^{-1}{\mathbf{A}}^{12}$ [7] and ${\mathbf{A}}_{22}^{-1}={\mathbf{A}}^{22}-{\mathbf{A}}^{21}{\left({\mathbf{A}}^{11}\right)}^{-1}{\mathbf{A}}^{12}$. Then, $${\mathbf{H}}^{-1}\mathbf{J}=\left[\begin{array}{c}{\mathbf{0}}\\ -{\mathbf{A}}_{22}^{-1}\end{array}\right]{\mathbf{Q}}_{\mathrm{c}}+\left[\begin{array}{c}{\mathbf{0}}\\ -\left({\mathbf{G}}^{-1}-{\mathbf{A}}_{22}^{-1}\right)\end{array}\right]{\mathbf{Q}}_{\mathrm{c}}=\left[\begin{array}{c}{\mathbf{0}}\\ {-\mathbf{G}}^{-1}\end{array}\right]{\mathbf{Q}}_{\mathrm{c}},$$ and $${\mathbf{J}}{\mathbf{^{\prime}}{\mathbf{H}}^{-1}}\mathbf{J}={\mathbf{J}}{\mathbf{^{\prime}}\left[\begin{array}{c}{\mathbf{0}}\\ {-\mathbf{G}}^{-1}\end{array}\right]{\mathbf{Q}}_{\mathrm{c}}}={\mathbf{Q}}_{\mathrm{c}}{\mathbf{^{\prime}}\left[-\begin{array}{cc}{\mathbf{A}}_{22}^{-1}{\mathbf{A}}_{21}& -\mathbf{I}\end{array}\right]\left[\begin{array}{c}{\mathbf{0}}\\ {-\mathbf{G}}^{-1}\end{array}\right]{\mathbf{Q}}_{\mathrm{c}}}={\mathbf{Q}}_{\mathrm{c}}{\mathbf{^{\prime}}{\mathbf{G}}^{-1}{\mathbf{Q}}_{\mathrm{c}}}.$$

Thus, the MME Eq. (3) can be written as:

4 $$\left[\begin{array}{cccc}{\mathbf{X}}^{\mathbf{\prime}}{\mathbf{R}}^{-1}\mathbf{X}& {\mathbf{0}}& {\mathbf{0}}& {\mathbf{X}}^{\mathbf{\prime}}{\mathbf{R}}^{-1}\mathbf{W}\\ {\mathbf{0}}& {\mathbf{Q}}_{\mathrm{c}}\mathbf{^{\prime}}{\mathbf{G}}^{-1}{\mathbf{Q}}_{\mathrm{c}}{\upsigma }_{a}^{-2}& -{\mathbf{Q}}_{\mathrm{c}}\mathbf{^{\prime}}{\mathbf{G}}^{-1}{\mathbf{Q}}_{2}{\upsigma }_{a}^{-2}& -{\mathbf{Q}}_{\mathrm{c}}^{\mathbf{\prime}}{\mathbf{F}}^{\mathbf{^{\prime}}}{\upsigma }_{a}^{-2}\\ {\mathbf{0}}& -{\mathbf{Q}}_{2}\mathbf{^{\prime}}{\mathbf{G}}^{-1}{\mathbf{Q}}_{\mathrm{c}}{\upsigma }_{a}^{-2}& {\mathbf{Q}}^{\mathbf{\prime}}{\mathbf{H}}^{-1}\mathbf{Q}{\upsigma }_{a}^{-2}+{\mathbf{S}}^{-1}& -{\mathbf{Q}}^{\mathbf{\prime}}{\mathbf{H}}^{-1}{\upsigma }_{a}^{-2}\\ {\mathbf{W}}^{\mathbf{\prime}}{\mathbf{R}}^{-1}\mathbf{X}& -\mathbf{F}{\mathbf{Q}}_{\mathrm{c}}{\upsigma }_{a}^{-2}& -{\mathbf{H}}^{-1}\mathbf{Q}{\upsigma }_{a}^{-2}& {\mathbf{W}}^{\mathbf{\prime}}{\mathbf{R}}^{-1}\mathbf{W}+{\mathbf{H}}^{-1}{\upsigma }_{a}^{-2}\end{array}\right]\left[\begin{array}{c}\widehat{\mathbf{b}}\\ \widehat{\mathbf{c}}\\ \widehat{\mathbf{g}}\\ {\widehat{\mathbf{a}}}_{d}\end{array}\right]=\left[\begin{array}{c}\mathbf{X}\mathbf{^{\prime}}{\mathbf{R}}^{-1}\mathbf{y}\\ {\mathbf{0}}\\ {\mathbf{0}}\\ \mathbf{W}\mathbf{^{\prime}}{\mathbf{R}}^{-1}\mathbf{y}\end{array}\right],$$

where $$\mathbf{F}=\left[\begin{array}{c}{\mathbf{0}}\\ -{\mathbf{G}}^{-1}\end{array}\right]$$ and ${\mathbf{Q}}_{2}$ are the rows of matrix $\mathbf{Q}$ pertaining to the genotyped animals. Thus, the coefficients to the regression effect $\widehat{\mathbf{c}}$ involve only functions of ${\mathbf{Q}}_{\mathrm{c}}$ and ${\mathbf{G}}^{-1}$, and no longer neither matrix $\mathbf{J}$ as in the MME Eqs. (2) and (3), nor the pedigree-based relationship matrix as in the MME Eq. (3).

Assuming that ${\mathbf{Q}}_{\mathrm{c}}\mathbf{^{\prime}}{\mathbf{G}}^{-1}{\mathbf{Q}}_{\mathrm{c}}$ is non-singular, MME Eq. (4) can be further simplified by absorption of the $\mathbf{c}$ effect to the other effects. Let $${\mathbf{C}}_{c,-c}=-{\upsigma }_{a}^{-2}\left[\begin{array}{ccc}{\mathbf{0}}& {{\mathbf{Q}}_{\mathrm{c}}\mathbf{^{\prime}}\mathbf{G}}^{-1}{\mathbf{Q}}_{2}& {\mathbf{Q}}_{\mathrm{c}}\mathbf{^{\prime}}\mathbf{F}\mathbf{^{\prime}}\end{array}\right]$$, i.e., the rows in the MME Eq. (4) coefficient matrix for the J factor effect $\widehat{\mathbf{c}}$ excluding columns having coefficients for $\widehat{\mathbf{c}}$. This can be rewritten as $${\mathbf{C}}_{c,-c}=-{\upsigma }_{a}^{-2}{\mathbf{Q}}_{\mathrm{c}}\mathbf{^{\prime}}{\mathbf{G}}^{-1}\left[\begin{array}{ccc}{\mathbf{0}}& {\mathbf{Q}}_{2}& \begin{array}{cc}{\mathbf{0}}& \mathbf{I}\end{array}\end{array}\right]=-{\upsigma }_{a}^{-2}{\mathbf{Q}}_{\mathrm{c}}\mathbf{^{\prime}}{\mathbf{G}}^{-1}{\mathbf{K}}_{\mathrm{Q}}$$, where $${\mathbf{K}}_{\mathrm{Q}}=\left[\begin{array}{ccc}{\mathbf{0}}& {\mathbf{Q}}_{2}& \begin{array}{cc}{\mathbf{0}}& \mathbf{I}\end{array}\end{array}\right]$$ has non-zero elements only at columns for the genetic groups (${\mathbf{Q}}_{2}$) and breeding values of genotyped animals ($\mathbf{I}$). Because the right-hand side values in the MME Eq. (4) corresponding to $\widehat{\mathbf{c}}$ are zero, the absorption changes only the coefficient matrix. The change due to the absorption is ${-\sigma }_{a}^{2}{\mathbf{C}}_{c,-c}\mathbf{^{\prime}}{\left({\mathbf{Q}}_{\mathrm{c}}\mathbf{^{\prime}}{\mathbf{G}}^{-1}{\mathbf{Q}}_{\mathrm{c}}\right)}^{-1}{\mathbf{C}}_{c,-c}=-{\upsigma }_{a}^{-2}{\mathbf{K}}_{\mathrm{Q}}\mathbf{^{\prime}}{\mathbf{G}}^{-1}{\mathbf{Q}}_{\mathrm{c}}{\left({\mathbf{Q}}_{\mathrm{c}}\mathbf{^{\prime}}{\mathbf{G}}^{-1}{\mathbf{Q}}_{\mathrm{c}}\right)}^{-1}{\mathbf{Q}}_{\mathrm{c}}\mathbf{^{\prime}}{\mathbf{G}}^{-1}{\mathbf{K}}_{\mathrm{Q}}={\upsigma }_{a}^{-2}{\mathbf{K}}_{\mathrm{Q}}\mathbf{^{\prime}}{\mathbf{K}}_{\mathrm{c}}{\mathbf{K}}_{\mathrm{Q}},$ where ${\mathbf{K}}_{\mathrm{c}}=-{\mathbf{G}}^{-1}{\mathbf{Q}}_{\mathrm{c}}{\left({\mathbf{Q}}_{\mathrm{c}}\mathbf{^{\prime}}{\mathbf{G}}^{-1}{\mathbf{Q}}_{\mathrm{c}}\right)}^{-1}{\mathbf{Q}}_{\mathrm{c}}\mathbf{^{\prime}}{\mathbf{G}}^{-1}$. Because matrix ${\mathbf{K}}_{\mathrm{Q}}$ operates only on the coefficients of the genotyped animals and the genetic groups through ${\mathbf{Q}}_{2}$, the MME Eq. (4) after absorption of the J factor effect is changed as:

5 $$\left[\begin{array}{ccc}\mathbf{X}\mathbf{^{\prime}}{\mathbf{R}}^{-1}\mathbf{X}& {\mathbf{0}}& \mathbf{X}\mathbf{^{\prime}}{\mathbf{R}}^{-1}\mathbf{W}\\ {\mathbf{0}}& \mathbf{Q}\mathbf{^{\prime}}{\mathbf{H}}_{{\varvec{J}}}^{*}\mathbf{Q}{\upsigma }_{a}^{-2}+{\mathbf{S}}^{-1}& -\mathbf{Q}\mathbf{^{\prime}}{\mathbf{H}}_{{\varvec{J}}}^{*}{\upsigma }_{a}^{-2}\\ \mathbf{W}\mathbf{^{\prime}}{\mathbf{R}}^{-1}\mathbf{X}& -{\mathbf{H}}_{{\varvec{J}}}^{*}\mathbf{Q}{\upsigma }_{a}^{-2}& \mathbf{W}\mathbf{^{\prime}}{\mathbf{R}}^{-1}\mathbf{W}+{\mathbf{H}}_{{\varvec{J}}}^{*}{\upsigma }_{a}^{-2}\end{array}\right]\left[\begin{array}{c}\widehat{\mathbf{b}}\\ \widehat{\mathbf{g}}\\ {\widehat{\mathbf{a}}}_{d}\end{array}\right]=\left[\begin{array}{c}\mathbf{X}\mathbf{^{\prime}}{\mathbf{R}}^{-1}\mathbf{y}\\ {\mathbf{0}}\\ \mathbf{W}\mathbf{^{\prime}}{\mathbf{R}}^{-1}\mathbf{y}\end{array}\right]$$

where $${\mathbf{H}}_{{\varvec{J}}}^{*}={\mathbf{A}}^{-1}+\left[\begin{array}{cc}{\mathbf{0}}& {\mathbf{0}}\\ {\mathbf{0}}& {\mathbf{G}}_{{\varvec{J}}}^{*}-{\mathbf{A}}_{22}^{-1}\end{array}\right]$$ and ${\mathbf{G}}_{{\varvec{J}}}^{*}={\mathbf{G}}^{-1}+{\mathbf{K}}_{\mathrm{c}}$. Thus, the J factors can be accounted in MME by changing the ${\mathbf{G}}^{-1}$ matrix without having to solve explicitly the regression effects $\mathbf{c}$ and to calculate the $\mathbf{J}$ matrix.

The absorption of the J factor effect in MME Eqs. (4) and (5) requires that the ${\mathbf{Q}}_{\mathrm{c}}\mathbf{^{\prime}}{\mathbf{G}}^{-1}{\mathbf{Q}}_{\mathrm{c}}$ matrix can be inverted. Matrix ${\mathbf{Q}}_{\mathrm{c}}\mathbf{^{\prime}}{\mathbf{G}}^{-1}{\mathbf{Q}}_{\mathrm{c}}$ is singular when $rank({\mathbf{Q}}_{\mathrm{c}})$ is less than the number of groups, i.e., there are linearly dependent groups. Observe also that ${\mathbf{G}}_{{\varvec{J}}}^{*}{\mathbf{Q}}_{\mathrm{c}}={\mathbf{0}}$ and that ${\mathbf{G}}_{{\varvec{J}}}^{*}\mathbf{G}{\mathbf{G}}_{{\varvec{J}}}^{*}={\mathbf{G}}_{{\varvec{J}}}^{*}$. Thus, the $\mathbf{G}$ matrix is by definition a generalized inverse of ${\mathbf{G}}_{{\varvec{J}}}^{*}$.

Note that the ${\mathbf{G}}_{{\varvec{J}}}^{*}$ matrix in the MME Eq. (5) is a computational result from absorbing the J factor effect, not an inverse of a genomic relationship matrix. In particular, ${\mathbf{G}}_{{\varvec{J}}}^{*}$ is singular as can be easily proved by observing that application of the Woodbury formula to invert ${\mathbf{G}}_{{\varvec{J}}}^{*}={\mathbf{G}}^{-1}-{\mathbf{G}}^{-1}{\mathbf{Q}}_{\mathrm{c}}{\left({\mathbf{Q}}_{\mathrm{c}}\mathbf{^{\prime}}{\mathbf{G}}^{-1}{\mathbf{Q}}_{\mathrm{c}}\right)}^{-1}{\mathbf{Q}}_{\mathrm{c}}\mathbf{^{\prime}}{\mathbf{G}}^{-1}$ will require the inversion of a singular matrix, i.e., a matrix of zeros.

### Special cases

An important special case in MME Eq. (5) is to have ${\mathbf{Q}}_{\mathrm{c}}={\mathbf{Q}}_{2}$, i.e., the same groups are used for centering and for the unknown genetic groups. Because now ${\mathbf{G}}_{{\varvec{J}}}^{*}{\mathbf{Q}}_{2}={\mathbf{G}}_{{\varvec{J}}}^{*}{\mathbf{Q}}_{\mathrm{c}}={\mathbf{0}}$, MME Eq. (5) can be written as:

6 $$\left[\begin{array}{ccc}\mathbf{X}\mathbf{^{\prime}}{\mathbf{R}}^{-1}\mathbf{X}& {\mathbf{0}}& \mathbf{X}\mathbf{^{\prime}}{\mathbf{R}}^{-1}\mathbf{W}\\ {\mathbf{0}}& \mathbf{Q}\mathbf{^{\prime}}\mathbf{E}\mathbf{Q}{\upsigma }_{a}^{-2}+{\mathbf{S}}^{-1}& -\mathbf{Q}\mathbf{^{\prime}}\mathbf{E}{\upsigma }_{a}^{-2}\\ \mathbf{W}\mathbf{^{\prime}}{\mathbf{R}}^{-1}\mathbf{X}& -\mathbf{E}\mathbf{Q}{\upsigma }_{a}^{-2}& \mathbf{W}\mathbf{^{\prime}}{\mathbf{R}}^{-1}\mathbf{W}+{\mathbf{H}}_{{\varvec{J}}}^{*}{\upsigma }_{a}^{-2}\end{array}\right]\left[\begin{array}{c}\widehat{\mathbf{b}}\\ \widehat{\mathbf{g}}\\ {\widehat{\mathbf{a}}}_{d}\end{array}\right]=\left[\begin{array}{c}\mathbf{X}\mathbf{^{\prime}}{\mathbf{R}}^{-1}\mathbf{y}\\ {\mathbf{0}}\\ \mathbf{W}\mathbf{^{\prime}}{\mathbf{R}}^{-1}\mathbf{y}\end{array}\right],$$

where $$\mathbf{E}={\mathbf{A}}^{-1}+\left[\begin{array}{cc}{\mathbf{0}}& {\mathbf{0}}\\ {\mathbf{0}}& -{\mathbf{A}}_{22}^{-1}\end{array}\right]$$ and ${\mathbf{G}}_{{\varvec{J}}}^{*}={\mathbf{G}}^{-1}+{\mathbf{K}}_{\mathrm{Q}}$ in ${\mathbf{H}}_{{\varvec{J}}}^{*},$ where ${\mathbf{K}}_{\mathrm{Q}}=-{\mathbf{G}}^{-1}{\mathbf{Q}}_{2}{\left({\mathbf{Q}}_{2}\mathbf{^{\prime}}{\mathbf{G}}^{-1}{\mathbf{Q}}_{2}\right)}^{-1}{\mathbf{Q}}_{2}\mathbf{^{\prime}}{\mathbf{G}}^{-1}$. Note that in MME Eq. (6) the genomic relationship matrix $\mathbf{G}$ makes no contribution to the coefficients involving the genetic group effects $\widehat{\mathbf{g}}$ because matrices $\mathbf{E}$ and $\mathbf{Q}$ are not functions of the $\mathbf{G}$ matrix.

Another special case is the original J factor model in [7] with ${\mathbf{Q}}_{\mathrm{c}}={\mathbf{1}}$, where $\widehat{\mathrm{c}}$ is a scalar valued regression effect. This will illustrate the MME in ssGBLUP when the original J factor of Fernando et al. [7] is used. Then, the absorption of the $\widehat{\mathrm{c}}$ effect in MME Eq. (4) gives MME Eq. (5) but with ${\mathbf{G}}_{{\varvec{J}}}^{*}={\mathbf{G}}^{-1}+{\mathbf{K}}_{1}$ and ${\mathbf{K}}_{1}=-{\mathbf{G}}^{-1}{\mathbf{11}}\mathbf{^{\prime}}{\mathbf{G}}^{-1}/\left(1\mathbf{^{\prime}}{\mathbf{G}}^{-1}{\mathbf{1}}\right)$. As before, ${\mathbf{G}}_{J}^{*}{\mathbf{1}}={\mathbf{0}}$, i.e., matrix ${\mathbf{G}}^{-1}$ has been replaced by ${\mathbf{G}}_{J}^{*}$, where the rows and columns sum to zero. However, when ${\mathbf{Q}}_{2}\ne {\mathbf{1}}$, the ${\mathbf{G}}_{{\varvec{J}}}^{*}{\mathbf{Q}}_{2}$ product can be different from zero. Thus, genomic data can influence coefficients of the genetic groups and the diagonal matrix for the genetic groups is $\mathbf{Q}\mathbf{^{\prime}}{\mathbf{H}}_{{\varvec{J}}}^{*}\mathbf{Q}{\upsigma }_{a}^{-2}+{\mathbf{S}}^{-1}$ as in MME Eq. (5).

### Computational considerations

In the derivations above, the genomic relationship matrix has the form $\mathbf{G}=\mathbf{Z}{\mathbf{D}}^{-1}\mathbf{Z}\mathbf{^{\prime}}$. When the number of genotyped animals is larger than the number of SNPs, this $\mathbf{G}$ matrix becomes singular. Common non-singular forms of the genomic relationship matrix are ${\mathbf{G}}_{\mathrm{C}}=\mathbf{G}+\mathbf{C}$, where the regularization matrix $\mathbf{C}$ is non-singular, easy to invert and independent of genomic information [16, 17]. Examples of such constrained genomic relationship matrices are ${\mathbf{G}}_{\varepsilon }=\mathbf{G}+\upvarepsilon \mathbf{I}$ and ${\mathbf{G}}_{w}=(1-\mathrm{w})\mathbf{G}+w{\mathbf{A}}_{22}$, where $\upvarepsilon$ is a small number and $w$ is the so-called residual polygenic proportion. There is always an equivalent single-step SNP-BLUP type of model for these ssGBLUP models [18]. Although the regularization matrix is not needed to avoid the singularity problem in single-step SNP-BLUP, a counterpart to the regularization matrix $\mathbf{C}$ is an independent random effect having a covariance matrix $\mathbf{C}$. In case $\mathbf{C}=w{\mathbf{A}}_{22}$, the genotyped animals have genomic and pedigree information weighted by a residual polygenic proportion, and in case $\mathbf{C}=\varepsilon \mathbf{I}$, an independent and identically distributed random effect is added to each genotyped animal. Thus, the earlier derivations for the J factor are valid and allow to account for any differences due to centering in $\mathbf{G}$ by the different allele coding by ssGBLUP models using ${\mathbf{G}}_{\varepsilon }$ or ${\mathbf{G}}_{w}$. However, note that differences in scaling, i.e., the $\mathbf{D}$ matrix, can lead to differences in GEBV. We will illustrate computational performance and consequences of the derivations using a ${\mathbf{G}}_{w}$ matrix in analysis of a small data set.

### Data

Approaches were tested using a subset of dairy cattle fertility data from Nordic Cattle Genetic Evaluation (NAV, Aarhus, Denmark). The data set is described in Matilainen et al. [14]. We considered only the two heifer fertility traits, i.e., nonreturn rate within 56 days after first service (NRR0) and days from first to last insemination (IFL0). The numbers of NRR and IFL0 observations were 6.5 million and 6.2 million, respectively. The pedigree included 5.4 million animals of which 33,969 were genotyped. There were 332 genetic groups which accounted for genetic level by breed, country of origin, and birth year. In the computations, we calculate the ${\mathbf{G}}_{{\varvec{J}}}^{*}$ matrix and solve MME Eq. (5) for different J factor models, where one of them has ${\mathbf{Q}}_{\mathrm{c}}$ equal to ${\mathbf{Q}}_{2}$. Computation of ${\mathbf{G}}_{{\varvec{J}}}^{*}$ requires that the ${\mathbf{Q}}_{\mathrm{c}}\mathbf{^{\prime}}{\mathbf{G}}^{-1}{\mathbf{Q}}_{\mathrm{c}}$ matrix is not singular. In other words, the genetic group matrix ${\mathbf{Q}}_{\mathrm{c}}$ cannot have linearly dependent rows/columns. The original groups defined and used in Matilainen et al. [14] led to a singular ${\mathbf{Q}}_{\mathrm{c}}\mathbf{^{\prime}}{\mathbf{G}}^{-1}{\mathbf{Q}}_{\mathrm{c}}$ matrix. Thus, for our study, we combined several adjacent birth year groups and reduced the number of groups from 332 to 232. The bulls were genotyped using the Illumina BovineSNP50 chip and the cows were genotyped using the BovineLD Bead Chip with the genotypes imputed to the 50K chip (Illumina Inc., San Diego, CA, USA).

### Study design

The residual polygenic proportion $w$ was 20% in the genomic relationship matrix ${\mathbf{G}}_{w}=(1-\mathrm{w})\mathbf{G}+w{\mathbf{A}}_{22}$, where $\mathbf{G}=\mathbf{Z}{\mathbf{D}}^{-1}{\mathbf{Z}}^{^{\prime}}$ was as in VanRaden’s method 1 [6]. Two sets of allele frequencies to center the $\mathbf{Z}$ matrix were tested. In the first approach, an allele frequency of 0.5 was used for all markers ($\mathbf{p}=0.5$). The second approach used base population allele frequencies which were estimated using the generalized least square (GLS) approach assuming a single breed [19] as implemented in the Bpop program [20]. The first approach is denoted by 101 and $\mathbf{G}$_101_ matrix, and the second approach by PvR1 and $\mathbf{G}$_PvR1_. Both $\mathbf{G}$ matrices used the same scaling factor $k=m/2$ where $m$ is equal to the number of markers. The same scaling factor $k$ allowed a scale independent comparison of the centering approaches.

The data were analyzed using three ssGBLUP models which had the same genetic groups. Two of the models had J factors, either as a single J factor (J1) or genetic group-based J factors (JQ), i.e., ${\mathbf{Q}}_{\mathrm{c}}={\mathbf{1}}$ or ${\mathbf{Q}}_{\mathrm{c}}={\mathbf{Q}}_{2}$, respectively. In the MME, the J factors and the genetic groups were either regression coefficients (reg) or pedigree groups after QP transformation as described earlier. Thus, we performed six ssGBLUP model analyses (Table 1). These models are referenced by the names QP, QPJ1, QPJQ, reg, regJ1, and regJQ. The term J1 will refer to both QPJ1 and regJ1, and JQ will refer to both QPJQ and regJQ. The reg model solved MME (2) and the QP model solved MME (5) with or without a J factor. The computational performance of the ssGBLUP approaches was measured by the number of iterations until convergence and computing time per iteration.

**Table 1:** Single-step model names and model differences in the mixed model equations

Name	J factor	Mixed model equations
reg	None	Regression effects for genetic groups
regJ1	${\mathbf{Q}}_{\mathrm{c}}={\mathbf{1}}$	Regression effects for genetic groups and one J factor
regJQ	${\mathbf{Q}}_{\mathrm{c}}={\mathbf{Q}}_{2}$	Regression effects for genetic groups and many J factors
QP	None	QP transformation of genetic groups
QPJ1	${\mathbf{Q}}_{\mathrm{c}}={\mathbf{1}}$	QP transformation of genetic groups and an absorbed J factor
QPJQ	${\mathbf{Q}}_{\mathrm{c}}={\mathbf{Q}}_{2}$	QP transformation of genetic groups and absorbed J factors

The models had either no J factors (None), one J factor (${\mathbf{Q}}_{\mathrm{c}}={\mathbf{1}}$) or multiple J factors as defined by the genetic groups (${\mathbf{Q}}_{\mathrm{c}}={\mathbf{Q}}_{2}$)

### Computations

The MiX99 software was used to solve MME to calculate GEBV using iteration on data and the PCG method [20] with a block diagonal preconditioner. The computations accounted for the inbreeding coefficients in ${\mathbf{A}}^{-1}$ and ${\mathbf{A}}_{22}$. The PCG method was assumed to be converged when ${\mathrm{C}}_{r}<{10}^{-7}$ where ${\mathrm{C}}_{r}$ is defined as the Euclidean norm of the difference between the right-hand side (RHS) of the MME and the one predicted by the current solutions relative to norm of the RHS:

$${\mathrm{C}}_{r}=\sqrt{\frac{\left(\mathbf{C}{\mathbf{s}}_{1}^{[k]}-\mathbf{r}\right)\mathrm{^{\prime}}\left(\mathbf{C}{\mathbf{s}}_{1}^{[k]}-\mathbf{r}\right)}{\mathbf{r}\mathrm{^{\prime}}\mathbf{r}},}$$

where ${\mathbf{s}}_{1}^{[k]}$ is the solution vector at round $k$, $\mathbf{C}$ is the MME coefficient matrix, and $\mathbf{r}$ is the MME right-hand side vector.

In the reg models, the regression coefficient matrices $\mathbf{W}\mathbf{Q}$ for the genetic groups and $\mathbf{W}\mathbf{J}$ for the J factors were precomputed and read from disk. The **Q** matrix was calculated based on pedigree information and this computation was fast (17s) using the RelaX2 program [22]. Two implementations for the $\mathbf{W}\mathbf{Q}$ matrix in solving MME Eq. (2) were tested. In the first approach, the $\mathbf{W}\mathbf{Q}$ matrix was considered as a dense matrix, and in the second approach, it was read to memory as a sparse matrix.

Two of the MME needed covariables in $\mathbf{W}\mathbf{J}$ for the J factors (regJ1 and regJQ). The values in the $\mathbf{J}$ matrix can be calculated using equality $$\mathbf{J}=\left[\begin{array}{c}-{\mathbf{A}}_{12}{\mathbf{A}}_{22}^{-1}\\ -\mathbf{I}\end{array}\right]{\mathbf{Q}}_{\mathrm{c}}=\left[\begin{array}{c}{\left({\mathbf{A}}^{11}\right)}^{-1}{\mathbf{A}}^{12}\\ -\mathbf{I}\end{array}\right]{\mathbf{Q}}_{\mathrm{c}}$$. Consider a column in ${\mathbf{Q}}_{\mathrm{c}}$ denoted as $\mathbf{v}$ and calculate $$\left[\begin{array}{c}{\mathbf{j}}_{1}\\ {\mathbf{j}}_{2}\end{array}\right]=\left[\begin{array}{c}{\left({\mathbf{A}}^{11}\right)}^{-1}{\mathbf{A}}^{12}\\ -\mathbf{I}\end{array}\right]\mathbf{v}$$, where vectors ${\mathbf{j}}_{1}$ and ${\mathbf{j}}_{2}$ have J factors for the non-genotyped and genotyped animals, respectively. A direct computational approach can be used in the calculation of the J factors [23]. However, we used standard genetic evaluation software in the calculation of the J factors by solving the following linear system of equations:

$$\left[\begin{array}{cc}{\uplambda \mathbf{A}}^{11}& {\uplambda \mathbf{A}}^{12}\\\uplambda {\mathbf{A}}^{21}& \mathbf{I}+\uplambda {\mathbf{A}}^{22} \end{array}\right]\left[\begin{array}{c}{\mathbf{j}}_{1}\\ {\mathbf{j}}_{2}^{*}\end{array}\right]=\left[\begin{array}{c}{\mathbf{0}}\\ -\mathbf{v}\end{array}\right],$$

where ${\mathbf{A}}^{\mathrm{ij}}$ is the sub-matrix $\mathrm{ij}$ of ${\mathbf{A}}^{-1}$, $\lambda =.001/.999$. Thus, the equations need to be solved for every J factor group. The equations were solved by MiX99 using the convergence limit ${\mathrm{C}}_{r}<{10}^{-9}$. The $\lambda$ ratio corresponds to the ratio of residual and genetic variances in an animal breeding MME. We used a small $\lambda$ value, which corresponds to having a high heritability. Consequently, the solutions ${\mathbf{j}}_{2}^{*}$ will be close to the right-hand side $\mathbf{-v}$. For safety’s sake, the precomputed values in $\mathbf{-v}$ were used as J factors for the genotyped animals instead of the ${\mathbf{j}}_{2}^{*}$ vector values.

## Results

Correlations of GEBV between the corresponding regression and QP transformation models for the genotyped and for all the animals were 100.00% for QP and QPJ1, but 99.99% for QPJQ, and the linear regression of GEBV by the QP model on the reg model had an intercept of 0 and a slope of 1. Thus, the regression and QP models resulted in the same GEBV. Correlations between GEBV solutions from different allele coding approaches were 100.00% between the J1 factor models and between the JQ models. Correlations of GEBV between the QP/reg and the J1 models were high, 100.00% for all animals and 99.99% for the genotyped animals. However, the JQ models gave GEBV that were distinctly different to those of the other models, the correlations ranged from 98.78 to 98.96% for all animals and from 83.95 to 85.74% for the genotyped animals. In other words, the use of either allele coding ($\mathbf{G}$_101_ or $\mathbf{G}$_PvR1_) did not affect GEBV results when a J factor was included in the model, and the full QP and reg models gave the same GEBV, as expected.

**Fig. 1:**
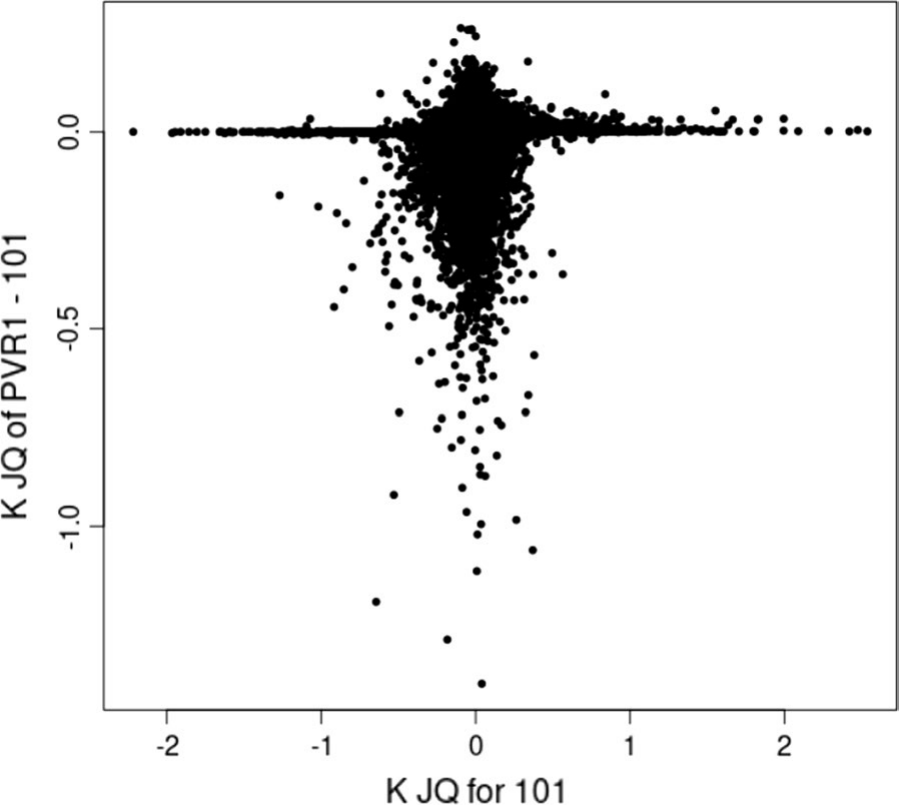
Non-zero values in the off-diagonal elements of the difference in the $\mathrm{K}$ matrix values (y axis) from VanRaden’s method 1 using base population allele frequencies (PvR1) or allele frequencies of 0.5 (101) for the $\mathrm{JQ}$ model. The x axis has the $\mathbf{K}$ matrix element value in the 101 $\mathbf{JQ}$ matrix

In the JQ models, using either the 101 or PvR1 allele coding, the GEBV were the same. Likewise, the ${\mathbf{G}}_{{\varvec{J}}}^{-1}$ matrix was the same irrespective of allele coding. However, the ${\mathbf{G}}^{-1}$ matrices were different by allele coding. The change needed in the ${\mathbf{G}}^{-1}$ matrix by allele coding to arrive to the ${\mathbf{G}}_{{\varvec{J}}}^{-1}$ matrix is measured by the ${\mathbf{K}}_{\mathrm{Q}}$ matrix. Figure 1 illustrates differences in the elements of the ${\mathbf{K}}_{\mathrm{Q}}$ matrix values between the two allele coding approaches. Values close to 0 on the x-axis mean that the elements have not changed much from the ${\mathbf{G}}_{101}^{-1}$ matrix. The change from ${\mathbf{G}}_{101}^{-1}$ to ${\mathbf{G}}_{{\varvec{J}}}^{-1}$ was mostly between – 1 and 1 with an average of 0. The y-axis shows differences in the ${\mathbf{K}}_{\mathrm{Q}}$ matrix elements of PvR1 minus 101 allele coding, i.e., elements of ${\mathbf{K}}_{\mathrm{Q},\mathrm{PvR}1}-{\mathbf{K}}_{\mathrm{Q},101}$. Values on the y-axis are mostly lower than 0. Thus, the ${\mathbf{G}}^{-1}$ matrix calculated by using base population allele frequencies had to be changed more than the ${\mathbf{G}}_{101}^{-1}$ matrix in order to arrive to the same ${\mathbf{G}}_{{\varvec{J}}}^{-1}$ matrix.

The average absolute diagonal and off-diagonal elements of the ${\mathbf{K}}_{\mathrm{Q},\mathrm{PvR}1}$ (${\mathbf{K}}_{\mathrm{Q},101}$) matrix were 8.99 × 10^–4^ (8.97 × 10^–4^) and 4.47 × 10^–2^ (4.42 × 10^–2^), respectively, with standard deviations of 2.58 × 10^–3^ (2.50 × 10^–3^) and 0.234 (0.229), respectively. One would have expected the base population-based genomic relationship matrix $\mathbf{G}$_PvR1_ to show smaller change than the $\mathbf{G}$_101_ matrix. A reason for ${\mathbf{G}}_{\mathrm{PvR}1}^{-1}$ to show slightly larger changes than ${\mathbf{G}}_{101}^{-1}$ can be due to the use of an incorrect scaling factor in $\mathbf{G}$_PvR1_ to allow the JQ models to reach the same GEBV. The used scaling factor $m/2$ is larger than the more correct $\sum_{l=1}^{m}2{p}_{l}\left(1-{p}_{l}\right)$, which would lead to ${\mathbf{K}}_{\mathrm{Q},\mathrm{PvR}1}$ that equals multiplying the current ${\mathbf{K}}_{\mathrm{Q},\mathrm{PvR}1}$ matrix by $\sum_{l=1}^{m}2{p}_{l}\left(1-{p}_{l}\right)\frac{2}{m}$ when there is no RPG component. This multiplier is 0.69 using marker information from our data.

**Fig. 2:**
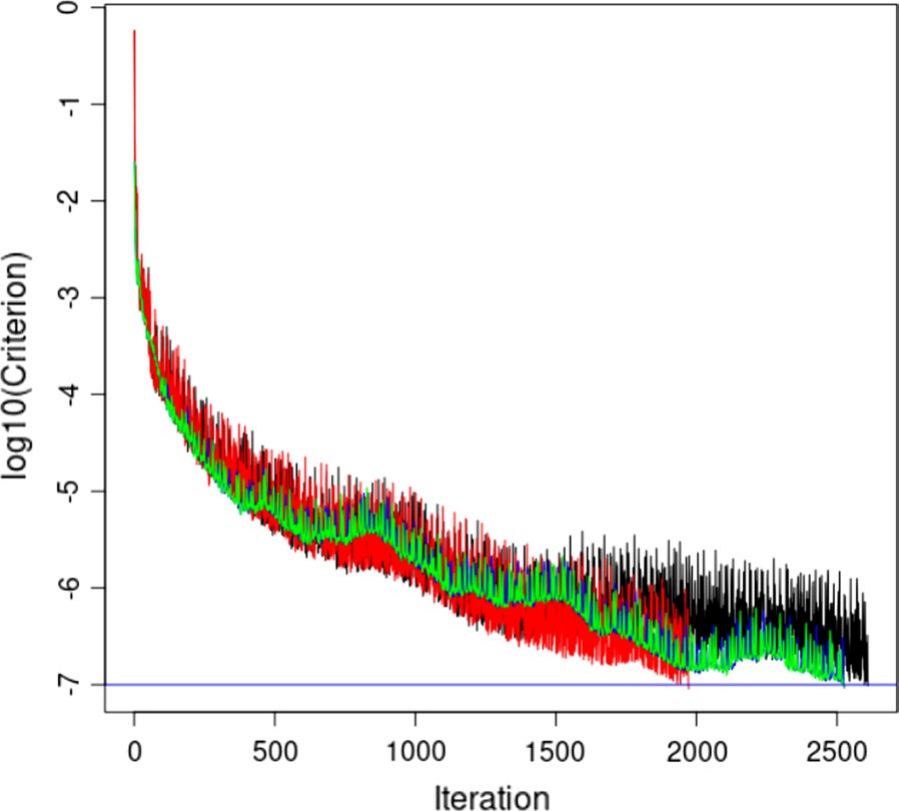
Logarithm of the convergence statistic ${\mathrm{C}}_{r}$ for the model that has JQ-based J factors. Black = PvR1 regression, red = 101 regression, blue = PvR1 QP + JQ, green = 101 QP + JQ

The number of iterations until convergence varied from 1531 to 2979 (Table 2). Note that the results are based on fertility data and a two-trait model for complex low heritable traits. The number of iterations varied more with the regression-based models than with the QP models. The reason is that the convergence criterion showed larger round-to-round changes in the regression models than in the QP models which led the convergence statistic to be reached more sporadically (Fig. 2). The larger variation in the convergence statistic with the regression models suggests that they could benefit from a better preconditioner. In all the analyses, the preconditioner was a block diagonal matrix with two-by-two trait blocks within each level of each effect. Apparently, compared to the QP models the reg models had larger off-diagonal values relative to the diagonal in the MME. Nevertheless, the regression model with J1 showed the smallest number of iterations until convergence.

**Table 2:** Number of iterations until convergence in single-step GBLUP when the centering of the markers for the genomic relationship matrix used base population allele frequencies (before the “/” sign) or an allele frequency of 0.5 for all markers (after the “/” sign)

Model	Groups	Groups + J1	Groups + JQ
Reg, dense	1999/2908	1646/1531	2979/2448
Reg, sparse	1991/2929	1647/1555	2961/2426
QP	1990/2134	2149/2051	2227/2207

Groups: the model had genetic groups; Groups + J1: the model had genetic groups and a single J factor; Groups + JQ: the model had genetic groups and group-wise J factors; reg, dense: regression coefficients for genetic groups and J factors in a dense matrix; reg, sparse: regression coefficients for genetic groups in a sparse matrix and J factors in a dense matrix; QP: genetic groups and J factors by QP transformation

Computing times per iteration for solving GEBV were shorter for the QP models than for the regression models (Table 3). The $\mathbf{W}$**Q** matrix was sparse with 6% of its elements being non-zero while the $\mathbf{W}\mathbf{J}$ matrix was dense with 95% of its elements being non-zero. When sparse matrix computations were used for the $\mathbf{W}$**Q** matrix, the regression models were almost as fast as the QP model except for JQ because of the dense $\mathbf{W}\mathbf{J}$ matrix computations.

**Table 3:** Computing time (seconds) per iteration to solve single-step GBLUP with base population allele frequencies in the genomic relationship matrix

Model	Groups	Groups + J1	Groups + JQ
reg, dense	3.18 (2.94)	3.09 (2.98)	4.31 (4.44)
reg, sparse	1.56 (1.49)	1.55 (1.59)	3.76 (4.07)
QP	1.30 (1.33)	1.38 (1.52)	1.51 (1.36)

Computing times in parentheses use an allele frequency of 0.5 for all markers

Groups: the model had genetic groups; Groups + J1: the model had genetic groups and a single J factor; Groups + JQ: the model had genetic groups and group-wise J factors; reg, dense: regression coefficients for genetic groups and J factors in a dense matrix; reg, sparse: regression coefficients for genetic groups in a sparse matrix and J factors in a dense matrix; QP: genetic groups and J factors by QP transformation

## Discussion

We used data on dairy cattle fertility and a two-trait model to illustrate the computational performance of the equivalent MME Eqs. (2) and (5). The observed differences in computing times per iteration (Table 3) are due to the number of multiplications in the MME coefficient matrix times a vector product that is needed in the PCG iteration. Differences in the numbers of multiplications per iteration for the QP and reg models in the computation of the MME coefficient matrix times a vector can be estimated. In MME Eq. (5) of the QP model, the difference in the number of multiplications is mostly due to the genetic groups related to the coefficient matrices ${\mathbf{Q}}_{2}\mathbf{^{\prime}}{\mathbf{G}}^{-1}{\mathbf{Q}}_{2}$, $\mathbf-{\mathbf{Q}}_{2}\mathbf{^{\prime}}{\mathbf{G}}^{-1}$ and $\mathbf-{\mathbf{G}}^{-1}{\mathbf{Q}}_{2}$ which were precomputed in our study. The precomputation allows a computationally simple implementation of the solver program where these precomputed matrices can be included into the ${\mathbf{G}}^{-1}$ matrix file and used with the same pedigree groups in many evaluations without the need for the solver to compute them for each evaluation. In the PCG iteration, the number of multiplications in the product of these matrices times a vector is $r\left(r+2{n}_{g}\right)$, where $r$ is the number of genetic groups and ${n}_{g}$ is the number genotyped animals. In MME Eq. (2) of the reg model, the difference in the numbers of multiplications is due to the regression coefficient matrices $\mathbf{W}\mathbf{J}$ and $\mathbf{W}\mathbf{Q}$, which are not present in the QP model. In order to estimate the number of multiplications, note that the implemented PCG iteration used computation by parts in iteration on data as described in Strandén and Lidauer [21]. For the genetic groups in the reg model, every PCG iteration required the product $\mathbf{Q}\mathbf{^{\prime}}\mathbf{W}\mathbf{^{\prime}}{\mathbf{R}}^{-1}{\mathbf{d}}_{y}$, where ${\mathbf{d}}_{y}=\mathbf{X}{\mathbf{d}}_{b}+\mathbf{W}\mathbf{J}{\mathbf{d}}_{c}+\mathbf{W}\mathbf{Q}{\mathbf{d}}_{g}+\mathbf{W}{\mathbf{d}}_{a}$. The terms with $\mathbf{W}\mathbf{J}$ and $\mathbf{W}\mathbf{Q}$ are not included in the QP model. Let us assume that the data file has the rows in the $\mathbf{J}$ and $\mathbf{Q}$ matrices corresponding to the observations, i.e., ${n}_{y}$ by $s$ matrix ${\mathbf{J}}_{{\varvec{W}}}=\mathbf{W}\mathbf{J}$ and ${n}_{y}$ by $r$ matrix ${\mathbf{Q}}_{{\varvec{W}}}=\mathbf{W}\mathbf{Q}$. Then, in the calculation of ${\mathbf{d}}_{y}$, the J factors (${\mathbf{J}}_{{\varvec{W}}}{\mathbf{d}}_{c}$) and the genetic groups (${\mathbf{Q}}_{{\varvec{W}}}{\mathbf{d}}_{g}$) require ${n}_{y}s$ and ${n}_{y}r$ multiplications, respectively, where ${n}_{y}$ is the number of observations. The multiplication ${\mathbf{Q}}_{{\varvec{W}}}\mathbf{^{\prime}}{\mathbf{R}}^{-1}{\mathbf{d}}_{y}$ requires ${n}_{y}r$ multiplications when the multiplications by the ${\mathbf{R}}^{-1}$ matrix are ignored. Thus, in total ${n}_{y}(s+2r)$ multiplications are required. When J factor computations are not present in the reg model, i.e., $s$ is equal to zero, the number of multiplications in the reg model ($=2{n}_{y}r$) is larger than in the QP model ($=r\left(r+2{n}_{g}\right)$) when ${n}_{y}>{n}_{g}+\frac{1}{2}r$. Thus, in practice, the number of multiplications per PCG iteration in a ssGBLUP model with QP is often smaller than in the corresponding reg model.

A sparse $\mathbf{W}\mathbf{Q}$ matrix allows to decrease the reg model solver computing time. Let us consider the differences in the number of multiplications in PCG between the QP and reg models. When there are no J factors and the $\mathbf{W}\mathbf{Q}$ matrix has a sparsity of $p$, the reg model has $2{n}_{y}rp$ multiplications not included in the QP model. Thus, the QP model has less multiplications than the reg model when ${n}_{y}>\frac{1}{p}{n}_{g}+\frac{1}{2p}r$. For example, assuming 5% of non-zeros in the $\mathbf{W}\mathbf{Q}$ matrix would have ${n}_{y}>20{n}_{g}+10r$, i.e., when the ratio between the number of genotyped and phenotyped animals is higher than the density of non-zeros in the **Q** matrix, the QP model has more multiplications than the reg model. However, in practice, the difference in computing time can be small when the number of genotyped animals is large. In this case, most of the computing time is due to the genomic relationship matrix.

The QP model has an added computational preprocessing cost due to the calculation of ${\mathbf{Q}}_{2}\mathbf{^{\prime}}{\mathbf{G}}^{-1}{\mathbf{Q}}_{2}$, $-{\mathbf{Q}}_{2}\mathbf{^{\prime}}{\mathbf{G}}^{-1}$ and $-{\mathbf{G}}^{-1}{\mathbf{Q}}_{2}$. The number of multiplications to calculate these matrices is $r{n}_{g}\left(r+{n}_{g}\right)$ when we note that the computational result from the two latter matrices (${\mathbf{G}}^{-1}{\mathbf{Q}}_{2}$) is an ${n}_{g}$ by $r$ matrix and can be used in the computation of the first matrix. This computational cost is small, because inversion of the $\mathbf{G}$ matrix is much more demanding since there are typically more genotyped animals than groups. Furthermore, these matrices are calculated only once but the numbers of multiplications given in the previous paragraphs are computed for each PCG iteration. Both of our genomic data sets were so small that we did not see any practical difference in computing time due to QP when making the augmented ${\mathbf{G}}^{-1}$ matrix having ${\mathbf{Q}}_{2}\mathbf{^{\prime}}{\mathbf{G}}^{-1}{\mathbf{Q}}_{2}$, $\mathbf-{\mathbf{Q}}_{2}\mathbf{^{\prime}}{\mathbf{G}}^{-1}$ and $\mathbf-{\mathbf{G}}^{-1}{\mathbf{Q}}_{2}$. The same was true when making the J1 adjustment to ${\mathbf{G}}^{-1}$. However, making the JQ adjustment to ${\mathbf{G}}^{-1}$ doubled the computing time. This increase in computing time was not significant compared to the total computing time.

Previous studies have recommended making the genomic relationship matrix compatible with the pedigree-based relationship matrix [3, 13]. The use of a J factor allows the calculation of allele-coding-free GEBV. Hence, the compatibility in a ssGBLUP model with a J factor means compatibility in scaling the marker matrix, which was the same in all our $\mathbf{G}$ matrices. Thus, while the J factor removes the necessity to center the marker matrix, proper scaling is still required. When centering and scaling use base population allele frequencies, the recommended scaling factor for a single breed in [6] is $\sum_{l=1}^{m}2{p}_{l}\left(1-{p}_{l}\right)$ instead of $m/2$ as used in our study. The use of a J factor will give GEBV that differ from those based on a $\mathbf{G}$ matrix where the base population allele frequencies have been estimated using the GLS approach as in this study. There is some evidence that a J factor can have a positive impact on the accuracy of prediction [8]. Correlations of GEBV for the genotyped animals between the JQ and J1 models were only about 85%, suggesting a notable difference in prediction ability. However, the accuracy of prediction in a multiple J factor model has not yet been studied. Thus, further work is necessary to assess the effect of a J factor on the predictability of GEBV but also the theoretical consequences of its use.

We used J factors to be able to calculate allele-coding-free GEBV, which parallels the work in [9] for the GBLUP and SNP-BLUP models. As in their study, the allele-coding-free GEBV calculated by the J1 and JQ models do not allow the computation of individual animal-based reliabilities that are allele-coding-free because the J factor effect cannot be included into the individual animal genetic variance term that is used as a denominator in the reliability equation. Diagonals of the $\mathbf{G}$ matrix from the J1 or JQ model depend on allele coding, likewise, the $\mathbf{H}$ matrix depends on allele coding even in the J1 and JQ models. Thus, computation of individual animal reliabilities of GEBV for the ssGBLUP model is relative to allele coding as in GBLUP and SNP-BLUP models even if allele-coding-free GEBV can be computed. When the J factor is considered random, then we can include the J factor into the $\mathbf{G}$ matrix and have well-defined genetic variances but then the model no longer produces allele-coding-free GEBV [10].

Tier et al. [24] suggested adding an implied founder animal as a genotyped animal in GBLUP. The founder animal will have its genotypes equal to the assumed allele frequencies in the founder population. The implied founder animal acts as the implied single genetic group for the base population animals in the pedigree-based relationship matrix $\mathbf{A}$. A desirable consequence of the Tier et al. [24] approach is that both the GEBV and their reliabilities are free from allele coding in GBLUP. In practice, the use of an additional random effect class allows the approach to achieve allele-coding-free reliabilities.

J factors can be random. Assuming that the J factor is a random effect allows inversion of the ${\mathbf{G}}_{{\varvec{J}}}^{-1}$ matrix. A random J factor will only slightly change the MME derived in the Methods section. Consider Model (1) but assume the J factor $\mathbf{c}$ to be random with expectation zero and variance ${\mathbf{S}}_{\mathrm{J}}{\sigma }_{a}^{2}$. The MME are like MME Eq. (2) but the diagonal block pertaining to the J factor is $\mathbf{J}\mathbf{^{\prime}}\mathbf{W}\mathbf{^{\prime}}{\mathbf{R}}^{-1}\mathbf{W}\mathbf{J}+{\mathbf{S}}_{{\varvec{J}}}^{-1}{\upsigma }_{a}^{-2}$. Use of the QP transformation gives MME Eq. (3) but with the matrix for the J factor equations as $\left(\mathbf{J}\mathbf{^{\prime}}{\mathbf{H}}^{-1}\mathbf{J}+{\mathbf{S}}_{{\varvec{J}}}^{-1}\right){\upsigma }_{a}^{-2}$. The simplification of the ${\mathbf{H}}^{-1}\mathbf{J}$ product leads to MME Eq. (4) but with the matrix for the J factor equations as $\left({\mathbf{Q}}_{\mathrm{c}}\mathbf{^{\prime}}{\mathbf{G}}^{-1}{\mathbf{Q}}_{\mathrm{c}}+{\mathbf{S}}_{{\varvec{J}}}^{-1}\right){\upsigma }_{a}^{-2}$. Absorption of the random J factor effect $\mathbf{c}$ to the other effects gives MME Eq. (5) except that now ${\mathbf{K}}_{\mathrm{c}}=-{\mathbf{G}}^{-1}{\mathbf{Q}}_{\mathrm{c}}{\left({\mathbf{Q}}_{\mathrm{c}}\mathbf{^{\prime}}{\mathbf{G}}^{-1}{\mathbf{Q}}_{\mathrm{c}}+{\mathbf{S}}_{{\varvec{J}}}^{-1}\right)}^{-1}{\mathbf{Q}}_{\mathrm{c}}\mathbf{^{\prime}}{\mathbf{G}}^{-1}$. The new matrix ${\mathbf{G}}_{{\varvec{J}},{\varvec{S}}}^{-1}={\mathbf{G}}^{-1}+{\mathbf{K}}_{\mathrm{c}}$ has an inverse unlike when the J factor was fixed: ${\mathbf{G}}_{J,S}=\mathbf{G}+{{\mathbf{Q}}_{\mathrm{c}}\mathbf{S}}_{\mathrm{J}}{\mathbf{Q}}_{\mathrm{c}}\mathbf{^{\prime}}$. Using different assumptions, Vitezica et al. [3] arrived to the same genomic relationship matrix, when ${\mathbf{Q}}_{\mathrm{c}}={\mathbf{1}}$ and ${\mathbf{S}}_{\mathrm{J}}=\alpha$ by assuming breeding values of the genotyped animals (${\mathbf{a}}_{2}$) to have mean µ and variance $\mathbf{G}{\sigma }_{a}^{2}$, i.e., ${\mathbf{a}}_{2}|\upmu \sim \mathrm{N}\left({\mathbf{1}}\upmu ,\mathbf{G}{\sigma }_{a}^{2}\right)$, where the mean $\upmu$ was assumed to be a random variable with expectation zero and variance $\alpha {\sigma }_{a}^{2}$, i.e., $\upmu \sim \mathrm{N}\left({0},\alpha {\sigma }_{a}^{2}\right)$, with $\alpha =\frac{\mathbf{1}}{{n}_{g}^{2}}\left({\mathbf{1}}\mathbf{^{\prime}}{\mathbf{A}}_{22}{\mathbf{1}}-{\mathbf{1}}\mathbf{^{\prime}}\mathbf{G}{\mathbf{1}}\right)$. Note that their MME Eq. (2) after absorption has ${\mathbf{G}}_{{\mathbf{1}},\alpha }^{-1}={\left(\mathbf{G}+{\mathbf{11}}\mathbf{^{\prime}}\alpha \right)}^{-1}$ which, after applying the Woodbury matrix identity, is ${\mathbf{G}}^{-1}-{\mathbf{G}}^{-1}{\mathbf{1}}{\left({\mathbf{1}}\mathbf{^{\prime}}{\mathbf{G}}^{-1}{\mathbf{1}}+{\alpha }^{-1}\right)}^{-1}{\mathbf{1}}\mathbf{^{\prime}}{\mathbf{G}}^{-1}$, i.e., the expression ${\mathbf{G}}_{{\varvec{J}},{\varvec{S}}}^{-1}={\mathbf{G}}^{-1}+{\mathbf{K}}_{\mathrm{c}}$ given above with ${\mathbf{Q}}_{\mathrm{c}}={\mathbf{1}}$ and ${\mathbf{S}}_{\mathrm{J}}=\alpha$.

Metafounders (MF) can be used to make the pedigree-based relationship matrices $\mathbf{A}$ and ${\mathbf{A}}_{22}$ more compatible with the $\mathbf{G}$ matrix [25] that is constructed using the allele frequency of 0.5 in VanRaden’s method 1. In the MF approach, unknown parents are assigned to MF or pseudo-individuals in the $\mathbf{A}$ matrix. MF increase the relationships in the pedigree and allow the assignment of a self-relationship to the MF. Thus, MF are like UPG but allow a related base population with non-zero inbreeding coefficients. Consequently, genetic groups are not included as effects in an MF model. Likewise, there is no justification for J factors in an MF model because a J factor would change the centering of the fixed 0.5 allele frequency in the $\mathbf{G}$ matrix.

We suggested that the number of J factors could equal the number of genetic groups, i.e., ${\mathbf{Q}}_{\mathrm{c}}={\mathbf{Q}}_{2}$. However, this can lead to collinear J factors with estimation problems similar to those for the estimation of many base population allele frequencies for the MF approach when applied to dairy cattle breeding (e.g., Kudinov et al. [26]). Their long pedigrees from the base population to the genotyped animals and the limited number of genotyped individuals in the pedigree can lead to poorly estimated base population allele frequencies. Consequently, the number of MF is typically smaller than the number of genetic groups in the analysis of the same data set using genetic groups. In this study, we had to limit the number of genetic groups such that they were fewer than in the original study by Matilainen et al. [14] due to the ${\mathbf{Q}}_{2}$ matrix having a lower rank than its number of columns when using the original number of genetic groups. So, in practice the absorption from MME Eq. (4) to MME Eq. (5) does not need to take all genetic groups into account but only those that are relevant to the genotyped animals such that the ${\mathbf{K}}_{\mathrm{Q}}={\mathbf{G}}^{-1}{\mathbf{Q}}_{2}{\left({\mathbf{Q}}_{2}\mathbf{^{\prime}}{\mathbf{G}}^{-1}{\mathbf{Q}}_{2}\right)}^{-1}{\mathbf{Q}}_{2}\mathbf{^{\prime}}{\mathbf{G}}^{-1}$ matrix can be computed. Thus, the number of J factor groups is unlikely to exceed the number of genetic groups for the complete pedigree and even a smaller number of J factor groups may give equally good breeding value predictions.

The genomic relationship matrix $\mathbf{G}$ does not contribute to the coefficients of the genetic group equations in MME Eq. (6) where it is assumed that the fixed J factors are composed using the same groups as the genetic groups. In fact, contributions to the UPG equations include only the terms due to the inverse pedigree relationship matrices ${\mathbf{A}}^{-1}$ and ${\mathbf{A}}_{22}^{-1}$. Thus, when the J factors and genetic groups have the same group structure for the genotyped animals, the genetic groups no longer have contributions due to genomic information. In the literature, the use of such MME to solve ssGBLUP breeding values has been advocated in some studies [27, 28]. These studies did not adjust the inverse genomic relationship matrix by the genetic group matrix ${\mathbf{Q}}_{2}$ to make and use the ${\mathbf{G}}_{{\varvec{J}}}^{*}$ matrix. However, the ${\mathbf{K}}_{{\varvec{c}}}$ matrix can have average values close to zero (Fig. 1). In our model, the ${\mathbf{K}}_{{\varvec{c}}}$ matrix can never have all its elements zero because this would lead to a singular ${\mathbf{Q}}_{\mathrm{c}}\mathbf{^{\prime}}{\mathbf{G}}^{-1}{\mathbf{Q}}_{\mathrm{c}}$ matrix. However, many elements in the absorbed term ${\mathbf{K}}_{{\varvec{c}}}=\mathbf-{\mathbf{G}}^{-1}{\mathbf{Q}}_{\mathrm{c}}{\left({\mathbf{Q}}_{\mathrm{c}}\mathbf{^{\prime}}{\mathbf{G}}^{-1}{\mathbf{Q}}_{\mathrm{c}}\right)}^{-1}{\mathbf{Q}}_{\mathrm{c}}\mathbf{^{\prime}}{\mathbf{G}}^{-1}$ can be close to zero. For example, in our case, the proportion of off-diagonal elements in the ${\mathbf{K}}_{{\varvec{c}}}$ matrix with an absolute value less than 10^–4^ was 75% for the 101 coding and 6% for the PvR1 coding. Thus, in some cases omitting the ${\mathbf{K}}_{{\varvec{c}}}$ term can be negligible or give even better predictability than the QP transformation model without a J factor.

The presented QP transformation for the J factor and consequent absorption yielding MME Eq. (5) is computationally simple for the single-step type of models where the inverse of the genomic relationship matrix ${\mathbf{G}}^{-1}$ is explicitly computed. When the number of genotyped animals is large, the $\mathbf{G}$ matrix will take too much memory and the ${\mathbf{G}}_{{\varvec{J}}}^{-1}$ matrix can no longer be calculated. A memory efficient single-step model alternative is ssGTBLUP [16], where the genomic relationship matrix is assumed to have the form $\mathbf{G}=\mathbf{Z}{\mathbf{D}}^{-1}\mathbf{Z}\mathbf{^{\prime}}+\mathbf{C}$ and its inverse is ${\mathbf{G}}^{-1}={\mathbf{C}}^{-1}-\mathbf{T}\mathbf{^{\prime}}\mathbf{T}$ with the rectangular matrix $\mathbf{T}$ of size equal to number of SNPs times number of genotyped animals. The absorption term ${\mathbf{K}}_{{\varvec{c}}}$ in MME Eq. (5) can be implemented for ssGTBLUP. Note that when ${\mathbf{G}}^{-1}={\mathbf{C}}^{-1}-\mathbf{T}\mathbf{^{\prime}}\mathbf{T}$ as in [16]:

$${\mathbf{K}}_{{\varvec{c}}}=-\left({\mathbf{C}}^{-1}-\mathbf{T}\mathbf{^{\prime}}\mathbf{T}\right){\mathbf{Q}}_{\mathrm{c}}{\left({\mathbf{Q}}_{\mathrm{c}}{^{\prime}}\left({\mathbf{C}}^{-1}-\mathbf{T}\mathbf{^{\prime}}\mathbf{T}\right){\mathbf{Q}}_{\mathrm{c}}\right)}^{-1}{\mathbf{Q}}_{\mathrm{c}}{^{\prime}}\left({\mathbf{C}}^{-1}-\mathbf{T}\mathbf{^{\prime}}\mathbf{T}\right)=-\left({\mathbf{C}}^{-1}-\mathbf{T}\mathbf{^{\prime}}\mathbf{T}\right){\mathbf{Q}}_{\mathrm{c}}{\left(\mathbf{L}\mathbf{L}\mathbf{^{\prime}}\right)}^{-1}{\mathbf{Q}}_{\mathrm{c}}{{^{\prime}}({\mathbf{C}}^{-1}-{\mathbf{T}}^{\mathbf{^{\prime}}}\mathbf{T})}=-{\mathbf{T}}_{\mathbf{K}}{{^{\prime}}{\mathbf{T}}_{\mathbf{K}}},$$

where ${\mathbf{T}}_{\mathbf{K}}={\mathbf{L}}^{-1}{\mathbf{Q}}_{\mathrm{c}}{^{\prime}}\left({\mathbf{C}}^{-1}-\mathbf{T}\mathbf{^{\prime}}\mathbf{T}\right)$ is an $s$ by ${n}_{g}$ matrix and $\mathbf{L}$ is an $s$ by $s$ matrix from Cholesky decomposition of ${\mathbf{Q}}_{\mathrm{c}}{^{\prime}}\left({\mathbf{C}}^{-1}-\mathbf{T}\mathbf{^{\prime}}\mathbf{T}\right){\mathbf{Q}}_{\mathrm{c}}$. Thus, ${\mathbf{G}}_{{\varvec{J}}}^{*}={\mathbf{C}}^{-1}-\mathbf{T}\mathbf{^{\prime}}\mathbf{T}-{\mathbf{T}}_{\mathbf{K}}\boldsymbol{^{\prime}}{\mathbf{T}}_{\mathbf{K}}$, which allows an easy and efficient PCG implementation. An alternative approach is to use MME Eq. (4). Then, as described for genetic groups in Koivula et al. [29], the $\mathbf{T}$ matrix can be augmented with $\mathbf{T}{\mathbf{Q}}_{\mathrm{c}}$ in order to have the $\mathbf{c}$ effect. However, some additional computations are needed because the $\mathbf{T}$ matrix does not contain the computations due to the ${\mathbf{C}}^{-1}$ matrix of ${\mathbf{G}}^{-1}$. The required computations due to the terms ${\mathbf{Q}}_{\mathrm{c}}\mathbf{^{\prime}}{\mathbf{C}}^{-1}{\mathbf{Q}}_{\mathrm{c}}$ and ${\mathbf{C}}^{-1}{\mathbf{Q}}_{\mathrm{c}}$ can be done by using precomputed matrices.

## Conclusions

The use of a J factor effect allows to compute GEBV in ssGBLUP and ssSNPBLUP that are independent of the allele coding used to center the marker matrix. We extended the single J factor regression to multiple group-based J factor regression effects. We used transformation in the MME of the ssGBLUP model to change the regression effect-based J factors to be correlated with genetic effects only. This showed a conceptual similarity of the J factors with the genetic groups which after a similar transformation can be used to augment the relationship matrix information. Furthermore, the transformation gave MME where the J factor coefficients do not need to be computed. When the number of J factor groups is large, solving the regression effect-based J factor MME can be computationally much more demanding than the transformed MME. Using the same regression coefficients for the J factor coefficients of the genotyped animals as for the genetic groups, we showed that the transformed MME in ssGBLUP no longer required the genomic relationship matrix to be accounted for in the genetic group equations when the J factor effects had been absorbed. We tested different J factor models for the analysis of a dairy fertility data set. We observed that GEBV were the same within a J factor model regardless of the allele coding approach as predicted by our derivations and that the QP transformed MME were computationally more efficient than the original regression-based MME. Further work is needed to assess predictability and proper individual reliability of GEBV when the model has a J factor.

## Data Availability

The phenotypic, pedigree, and original SNP data are property of the Nordic cattle breeding organizations, Viking Genetics (Randers, Denmark) and Nordic Cattle Genetic Evaluation Ltd (NAV, Aarhus, Denmark. Data are not for public distribution.
